# SPATA12 and Its Possible Role in DNA Damage Induced by Ultraviolet-C

**DOI:** 10.1371/journal.pone.0078201

**Published:** 2013-10-18

**Authors:** Yunsheng Zhang, Lifang Yang, Yiting Lin, Zhuoxian Rong, Xiaowen Liu, Dan Li

**Affiliations:** 1 Department of Life Science, College of Biology, Hunan University, Changsha, P. R. China; 2 Cancer Research Institute, Xiangya School of Medicine, Central South University, Changsha, P. R. China; St. Georges University of London, United Kingdom

## Abstract

Our previous studies indicated that SPATA12, a novel spermatogenesis-associated gene, might be an inhibitor involved in spermatogenesis and tumorigenesis. To obtain a better understanding of the functions of SPATA12, a yeast two-hybrid screening system was used to search for interacting proteins, and chromodomain helicase DNA binding protein 2 (CHD2) was successfully identified. Bimolecular fluorescence complementation (BiFC) and subcellular co-localization assays further suggested a possible interaction between SPATA12 and CHD2 in the nuclei. CHD2 is known to be involved in the later stage of the DNA damage response pathway by influencing the transcriptional activity of p53. Thus, our hypothesis is that SPATA12 might play a role in DNA damage signaling. Western blotting results showed that SPATA12 expression could be induced in ultraviolet-C (UV-C) irradiated cells. Through reporter gene assays and the activator protein-1 (AP-1) decoy oligodeoxynucleotide method, we demonstrated that *SPATA12* promoter activity could be up-regulated in response to UV-C radiation exposure and an AP-1 binding site in the *SPATA12* promoter may have a role in transcriptional regulation of *SPATA12*. Using colony formation and host cell reactivation assays, it was demonstrated that SPATA12 might lead to inhibition of cellular proliferation in UV-C-irradiated DNA damage. Furthermore, SPATA12 was transfected into H1299, MCF-7 and HeLa cells, and flow cytometry (FCM) results suggested that there are some biological association between SPATA12 and p53 in UV-C-irradiated DNA damage. In addition, we investigated whether SPATA12 could up-regulate the expression of p53. Taken together, these findings indicate that SPATA12 could be induced under UV-C stress. During DNA damage process, AP-1 involves in the transcriptional up-regulation of *SPATA12* in response to UV-C radiation and p53 involves in growth inhibitory effects of SPATA12 on UV-C irradiated cells.

## Introduction

Maintaining genomic integrity is a critical requirement for normal cell growth and development. UV radiation, genotoxic chemicals and ionizing radiation are potential sources for cellular DNA damage. The consequences of DNA damage are diverse and adverse, including DNA base modifications, crosslinks and single and double strand breaks (SSBs and DSBs) [1]. The inability to sense and respond to genotoxins leads to various disorders in mammals, such as cell death, genomic instability or malignant transformation [2]. Thus, it is important to understand how cells respond to and attempt to repair DNA damage.

Emerging evidence indicates that various modulations to chromatin structure are centrally important to many aspects of the DNA damage response (DDR) [3]. Genetic studies have revealed that mutant forms of histone modifying proteins and chromatin remodelers often show sensitivity to genetic stress [4]. Chromatin remodeling complexes such as the SWI-SNF family assist in double strand break repair specifically through the homologous recombination pathway. Defects in chromatin complexes result in poor cellular responses to DNA double strand breaks, resulting in an accumulation of genomic alterations and the potential for cancer development [3]. Recently, chromodomain helicase DNA binding protein 2 (CHD2), a SNF superfamily protein, was identified as having transcriptional regulatory activity and found to be directly involved in DNA damage responses by affecting the transcriptional activity of p53[5-7]. This clearly implicates CHD2 as a novel chromatin-remodeling factor required for genomic stability maintenance.

Cell viability in response to DNA damage relies not only on chromatin remodeling but also on a global transcriptional program to facilitate DNA repair or trigger cell cycle arrest and cellular apoptosis. As a guardian of the genome, p53 mediates the response to various stress signals and plays a crucial role in the DDR signaling cascade [8-10]. The p53 protein can be rapidly induced by multiple types of DNA damage, and induced p53 functions as a transcription factor for downstream genes involved in pathways of cell cycle regulation, apoptosis and/or DNA repair [9]. Thus, activation of the p53 pathway upon genotoxic stress could form a critical barrier against genomic lesions and tumor development [11]. 

The spermatogenesis-associated gene 12 gene (SPATA12), mapped to chromosome 3p14, was identified in our previous study and shown in seminiferous tubules of human adult testis—more precisely, in spermatocytes, spermatids and spermatozoa[12]. Our previous studies also implicated that SPATA12 might be an inhibitor of tumorigenesis [13]. However, the precise function of SPATA12 is unclear. One method to characterize the function of a protein is through the identification of the proteins with which it interacts. The yeast two-hybrid screening system is a powerful genetic strategy for this purpose. In the present study, we used a yeast two-hybrid system to search for proteins interacting with SPATA12 and identified CHD2 as a potential interactor. We also showed that the expression of SPATA12 can be induced by UV-C radiation and SPATA12 may lead to inhibition of cellular proliferation after DNA damage. Additionally, our results suggested that both AP-1 and p53 involve in pathway of SPATA12 in DNA damage.

## Results

### Identification of CHD2 as an interacting protein of SPATA12

**Figure 1:**
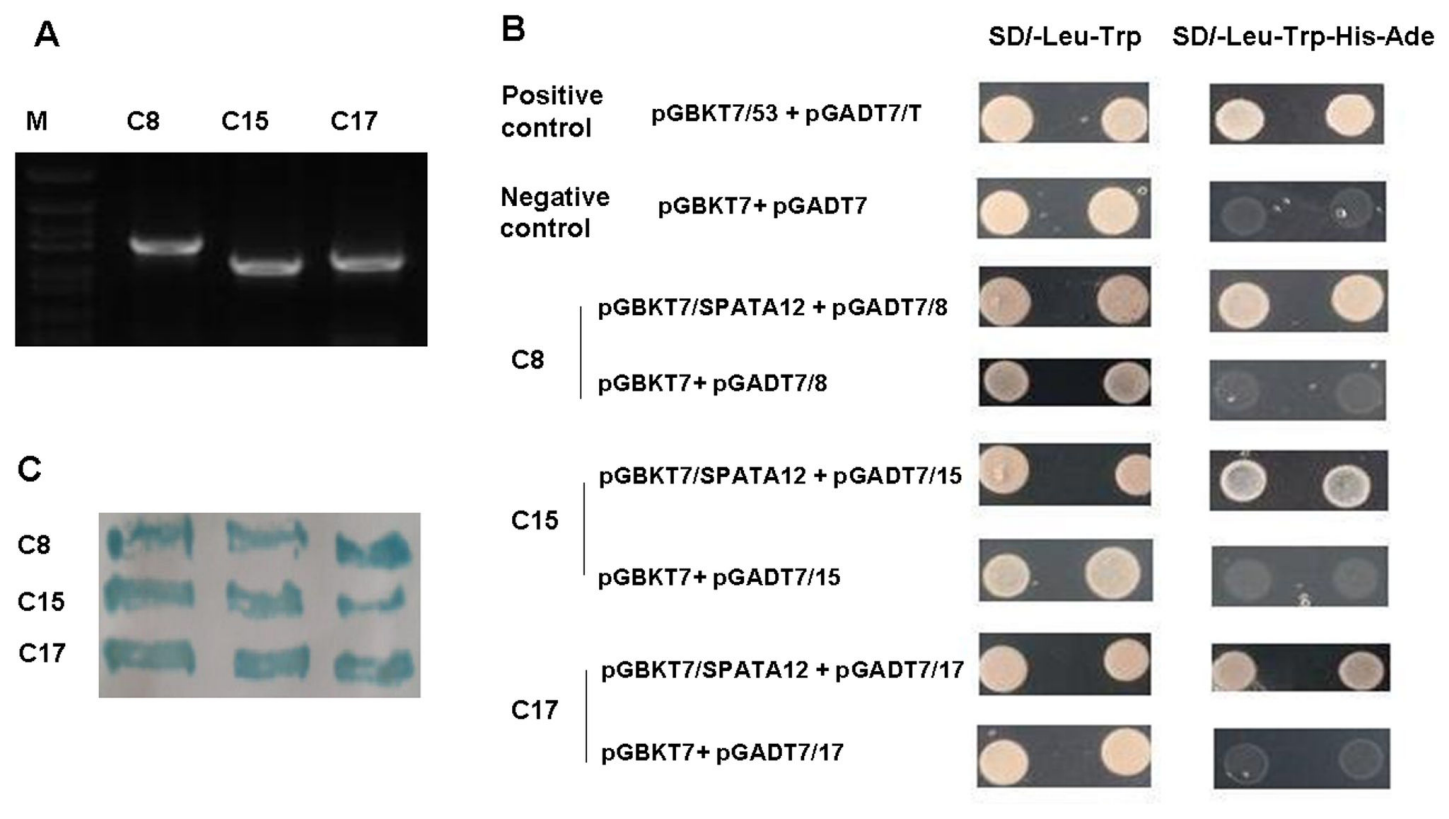
Identification of the protein interacting with SPATA12. A: Positive colony PCR of testis cDNA library. C8, C15 and C17 represent the inserts of pGADT7-Rec amplified by PCR. B: Interaction of SPATA12 and C8, SPATA12 and C15, and SPATA12 and C17 were verified by re-transformation into yeast cells. The yeast containing pGBKT7/53 was used as a positive control and yeast cells transformed with pGBKT7 were used as the negative control. C: The blue reaction indicated expression of β-galactosidase (lacZ product). C17 represents CHD2; C15 represents TFIIA; C8 represents an unknown sequence.

The yeast two-hybrid screening data showed that the positive colonies, including C8, C15 and C17(Figure 1A), were co-expressed with SPATA12 and promoted expression of the reporter genes, resulting in cells able to grow on media lacking tryptophan, leucine, adenine and histidine, compared with the negative control pGBKT7 and positive control pGBKT7/53 (Figure 1B). And this result was further verified by β-galactosidase report gene assay (Figure 1C). By sequencing (data not shown), C15 was verified to be homo sapiens general transcription factor IIA (TFIIA) and C17 was verified to be homo sapiens chromodomain helicase DNA binding protein 2 (CHD2), while C8 represents an unknown sequence. 

**Figure 2:**
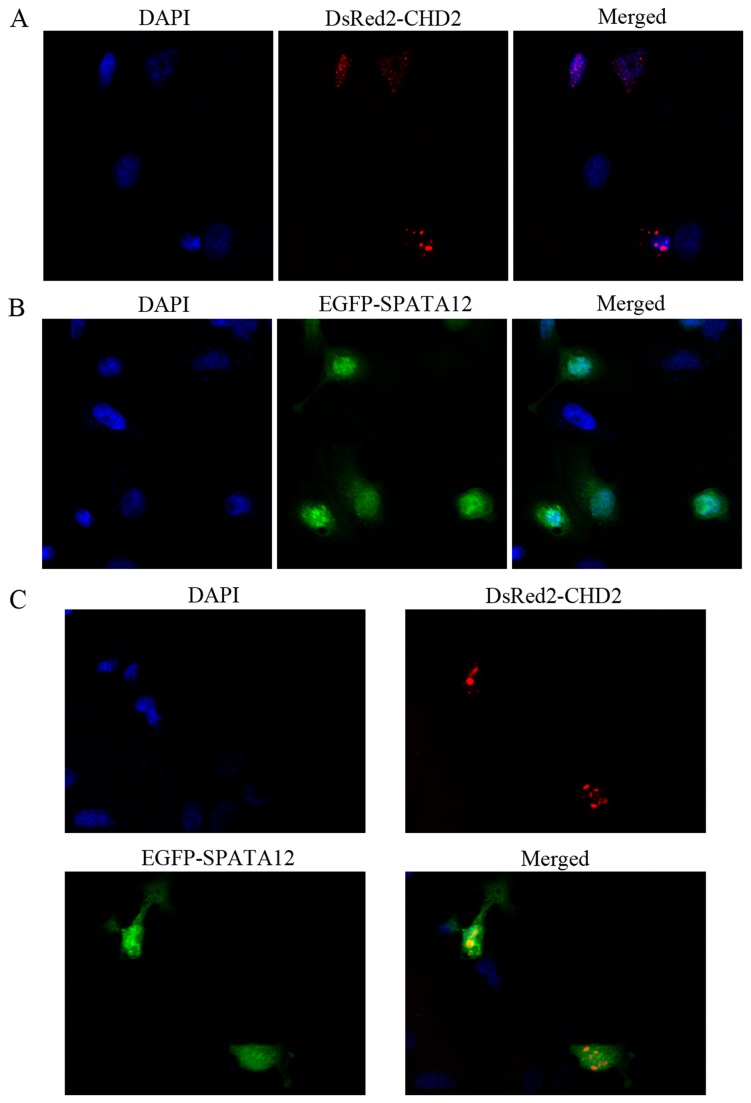
Subcellular distribution of SPATA12 and CHD2. A: Cells were transfected with the pEGFP-SPATA12 plasmid; green indicates the nuclear localization of exogenous SPATA12 (×400). B: Cells were transfected with the pDsRed-CHD2 plasmid; red illustrates the nuclear localization of exogenous CHD2 (×400). C: Cells were co-transfected with pEGFP-SPATA12 and pDsRed-CHD2 plasmids; yellow represents merged co-localization of SPATA12 and CHD2 in nuclei (×400).

In this study, we focused on the interaction between CHD2 and SPATA12. As shown in Figure 2A-C, the expression signal of pEGFP-SPATA12 was mainly located in the nuclei, while pDsRed-CHD2 was confined only to the nuclei. The yellow image represents the merged partial nuclear co-localization of SPATA12 and CHD2 in cells. 

**Figure 3:**
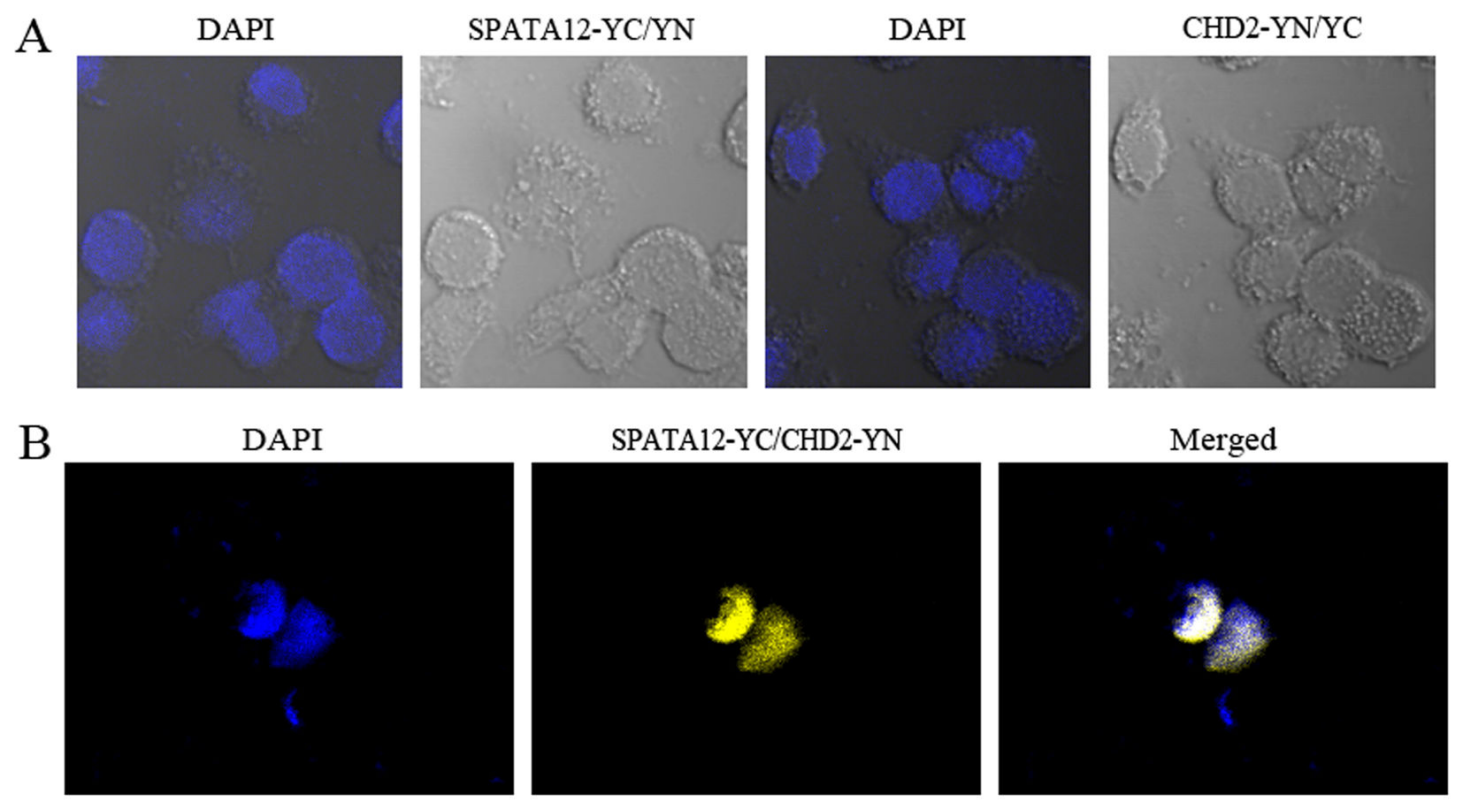
The interaction of SPATA12 and CHD2 verified by bimolecular fluorescence complementation (BiFC) assay. A: Cells co-transfected with pcDNA3.1(+)-SPATA12-YC and pcDNA3.1(+)-YN, pcDNA3.1(+)-CHD2-YN and pcDNA3.1-YC were served as negative controls (×400). B: Cells were co-transfected with pcDNA3.1(+)-CHD2-YN and pcDNA3.1(+)-SPATA12-YC plasmids; yellow signal represents interaction of SPATA12 and CHD2 in nuclei (×400).

In order to further support this potential spatial interaction of SPATA12 and CHD2 in nuclei, bimolecular fluorescence complementation (BiFC) assay in cells were used and cells were co-transfected with pcDNA3.1-SPATA12-YC and pcDNA3.1-CHD2-YN. As shown in Figure 3B, the yellow image indicated the interaction between SPATA12 and CHD2 in the nuclear. And cells co-transfected respectively with pcDNA3.1-SPATA12-YC and pcDNA3.1-YN, pcDNA3.1-CHD2-YN and pcDNA3.1-YC were served as negative controls (Figure 3A).

 These results suggested the possibility of a functional link between SPATA12 and CHD2.

### Establishment of a cellular DNA damage model induced by UV-C

**Figure 4:**
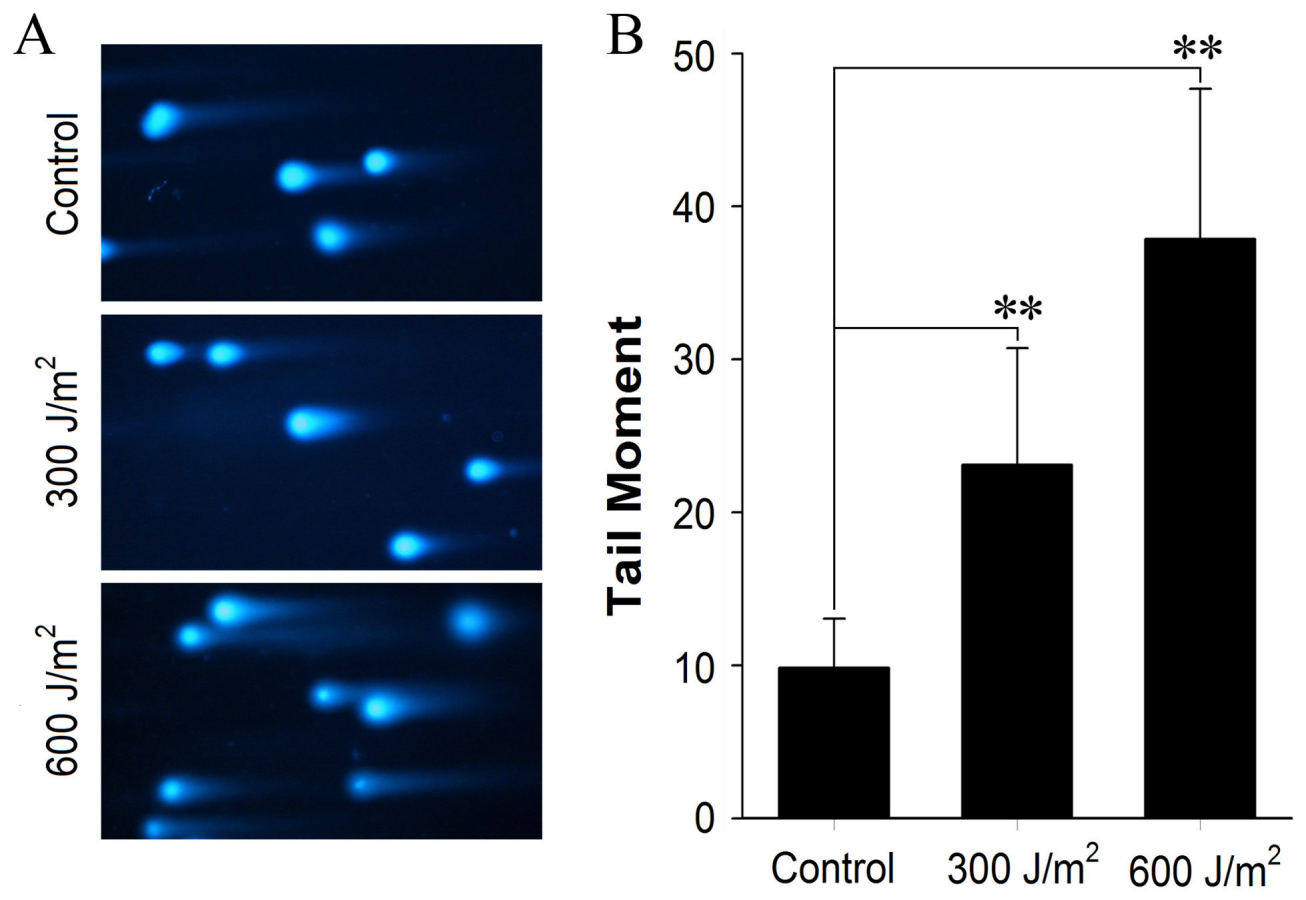
The analysis of chromosomal DNA integrity after UV-C irradiation. DNA damage (tail moment) induced by various does of UV-C radiation was detected by the neutral comet assay in HeLa cells. A: Representative images from the neutral comet assay (×200). B: UV-C-induced DNA damage as indicated by tail moment. **p<0.01 versus control group by *t*-test.

Studies indicated that the CHD2 protein plays a role in mediating genomic stability through DDR. Based on the interaction between SPATA12 and CHD2, we hypothesized that SPATA12 might be involved in DDR as well. Here, we established a cellular DNA damage model induced by ultraviolet-C (UV-C) radiation. HeLa cells were directly exposed to various doses of UV-C (254 nm), and 3-(4.5-dimethylthiazol-2-yl)-2,5-diphenyl tetrazolium bromide (MTT) assay showed a significant decrease in cell viability with increasing doses of 300 J/m^2^, 600 J/m^2^, 900 J/m^2^ and more UV-C radiation for 60 mins (Figure S1A). FCM analysis of HeLa cells showed a typical apoptosis peak at 600 J/m^2^ (Figure S1B, C), and double-staining indicated chromatin condensation at 600 J/m^2^ UV-C (Figure S1D). Next, the neutral comet assay was performed to further examine the integrity of chromosomal DNA after UV-C irradiation. Significant DSBs, indicated by the tail moment of the neutral comet assay, were observed in HeLa cells at 300 J/m^2^ and 600 J/m^2^ of UV-C radiation compared with control cells (Figure 4). Based on the above results, in subsequent experiments, the dose of UV-C radiation was set at 600 J/m^2^ and the processing time was set to 60 min to investigate the biological effects on SPATA12 at the cellular level.

### The expression of SPATA12 is induced by UV-C radiation

**Figure 5:**
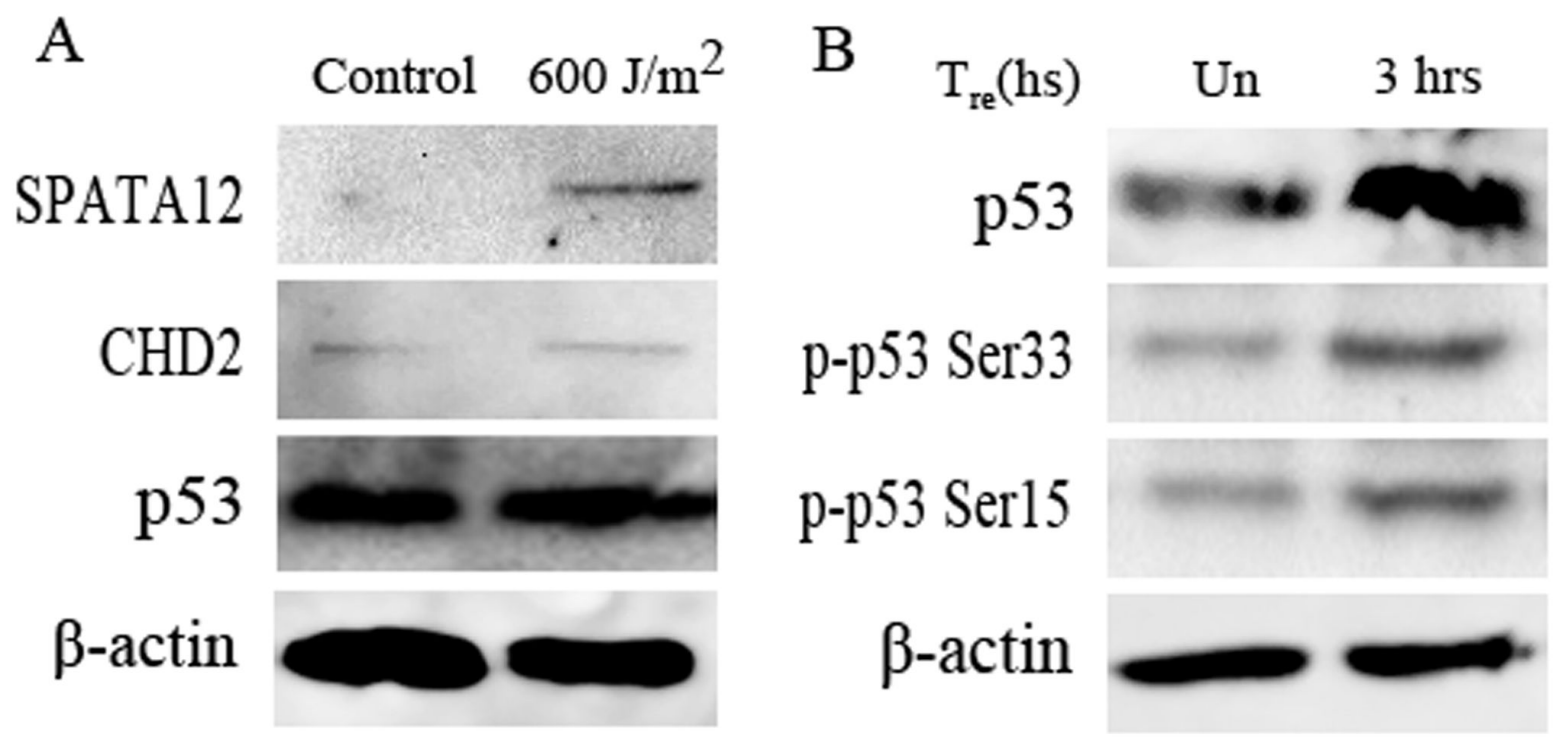
The expression of SPATA12 was induced by UV-C radiation. A: The protein levels of SPATA12, CHD2 and p53 in UV-C radiated cells were detected by western blot, respectively. β-actin was used as the normalization control. B: The protein levels of p53 and p-p53 (Ser 15 and Ser 33) in cells were detected 3 hours post UV-C-irradiation, respectively. β-actin was used as normalization control.

To explore the involvement of SPATA12 in DDR, we first detected whether SPATA12 could bind with CHD2 in nuclei in DNA damage conditions. The data showed that the yellow signals of protein interaction could be investigated in nuclei in UV-C-irradiated cells (Figure S2). Next, we detected whether SPATA12 could be induced in UV-C-irradiated cells. The results of real-time RT-PCR showed that *SPATA12* and *CHD2* could be induced in UV-C radiated DNA damage at mRNA level (Figure S3), while *p53* mRNA was gradually decreased (Figure S3). At the protein level, the expression of SPATA12 was induced after irradiation (Figure 5A), suggesting that SPATA12 could respond to the UV-C-induced DNA damage signal. There was no obvious change in the protein levels of CHD2 and p53 after UV-C treatment (Figure 5A). However, after recovery for 3 hours in UV-C treated cells, the protein levels of p53 and phosphorylation of the p53 protein at ser15 and ser33 were up-regulated (Figure 5B).

### AP-1 involves in the transcriptional up-regulation of *SPATA12* in response to UV-C radiation

**Figure 6:**
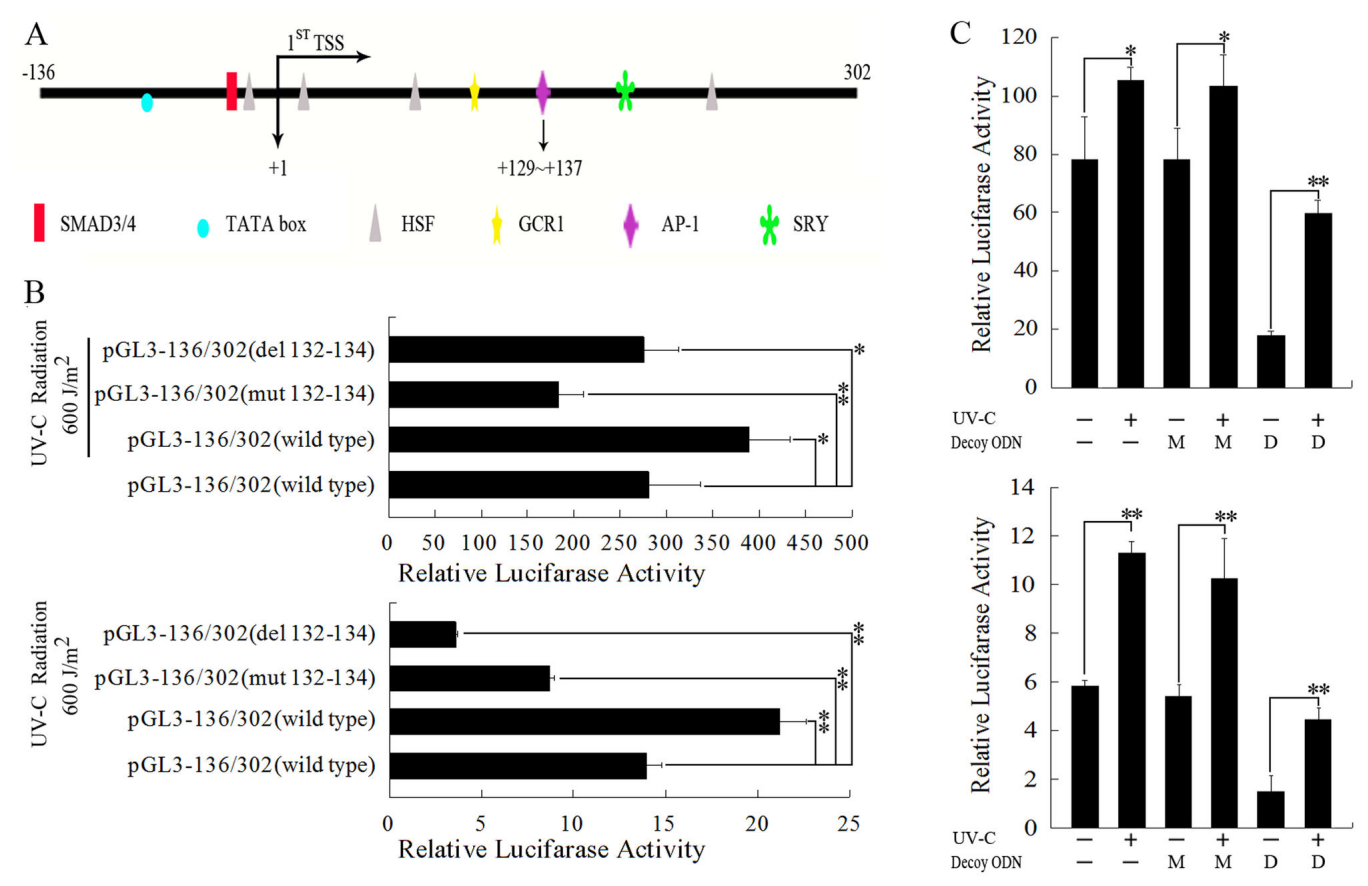
AP-1 involves in the transcriptional up-regulation of *SPATA12* in response to UV-C radiation. A: A schematic representation of the 5'-regulated region of SPATA12 gene. B: The AP-1 binding site involves in transcriptional regulation of SPATA12 in response to UV-C radiation. Renilla and firefly luciferase reporter genes combined with the wild-type, point mutation and deletion mutation versions of the SPATA12 promoter construct were co-transfected into cells prior to UV-C irradiation. The data are the means ± SD (n=3). **p<0.01, *p<0.05 versus the control groups by *t*-test. Top: MCF-7 cell lines. Bottom: HeLa cell lines. C: Relative luciferase activity analysis of SPATA12 promoter construct (pGL3-136/302, wild-type) using the dual luciferase reporter gene system after AP-1 decoy ODN treatment in cells. Renilla and firefly luciferase reporter genes, combined with AP-1 decoy ODNs or mismatched decoy ODNs, respectively, were co-transfected into cells prior to UV-C irradiation.The mismatched decoy ODNs served as negative controls. The data are the means ± SD (n=3). In blank control groups (cells without decoy ODN), **p<0.01, *p<0.05 versus the control groups by *t*-test. M indicates mismatched AP-1 decoy ODN; D indicates AP-1 decoy ODN. Top: MCF-7 cell lines. Bottom: HeLa cell lines.

Our previous study demonstrated that there are several important transcription factor binding sites, including an activator protein-1 (AP-1) binding site, in the *SPATA12* core promoter region (Figure 6A) [14]. In order to investigate the changes of *SPATA12* promoter activity in response to UV-C radiation and possible role of the AP-1 binding site during this process, the point mutation construct pGL3-136/302 (mut 132–134) and the deletion construct pGL3-136/302 (del 132–134) were generated to disrupt AP-1 binding (Figure 6A) [14]. Figure 6B showed that through this reporter system, the relative luciferase activity of the *SPATA12* core promoter (pGL3-136/302 wt, including the AP-1 binding site) was significantly increased after UV-C irradiation. However, under UV-C stress, the relative luciferase activity of the *SPATA12* core promoter with deleted or mutated AP-1 binding sites was significantly decreased in both HeLa and MCF-7 cells (Figure 6B). On the other hand, when AP-1 was inhibited by AP-1 decoy ODNs, we also observed an obvious decrease in the relative luciferase activity of the *SPATA12* core promoter in UV-C irradiated cells compared with both blank groups and control ODN groups (Figure 6C). The results indicated that: (1) the activity of *SPATA12* core promoter could be up-regulated by UV-C radiation; (2) the AP-1 binding site may involve mediating the UV-C induced up-regulation of *SPATA12* promoter activity. However, the results of Figure 6C also suggested a possibility that other transcriptional factors’ role in regulation of *SPATA12* after DNA damage await discovery.

### SPATA12 induces cell growth inhibition after UV-C radiation-induced DNA damage

**Figure 7:**
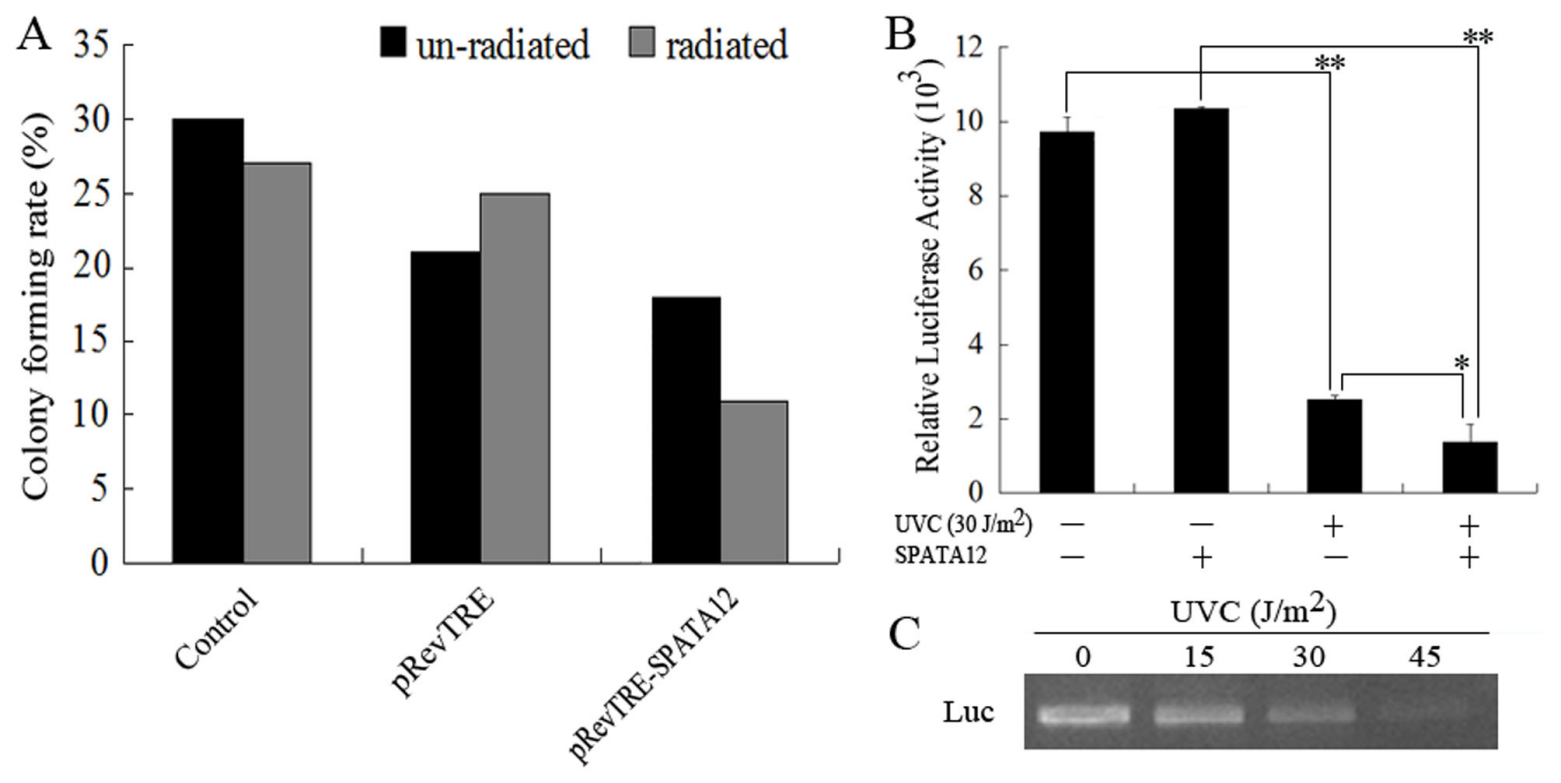
SPATA12 leads to the inhibition of cell-proliferation after UV-C irradiation. A: The capacity for migration of HeLa cells transfected with SPATA12 was evaluated by colony formation assay after UV-C irradiation. B: The effect of SPATA12 on DNA damage repair was examined by HCR assay in HeLa cells. **p<0.01, *p<0.05 versus the control groups by *t*-test. C: PCR analysis of UV-C-damaged plasmids. The data represent the mean ± SD (n=3).

 Colony formation ability and host cell reactivation (HCR) assays were performed to understand the possible functions of SPATA12 in DDR. The colony formation data showed that SPATA12 might suppress cell proliferation compared with vector controls after UV-C irradiation at 600 J/m^2^ (Figure 7A). In the HCR experiment, the pGL-3 control plasmid containing a luciferase reporter was UV-C irradiated at 15, 30, 45 J/m^2^ to generate DNA adducts that were confirmed by PCR analysis (Figure 7C). The damaged reporter plasmids were then transfected into HeLa-SPATA12 cells, and the luciferase activity of the reporter was analyzed. Results showed that the luciferase activity was reduced in HeLa-SPATA12 cells compared with control HeLa cells (Figure 7B), illustrating that SPATA12 may induce cell growth inhibition after DNA damage.

### p53 involves in growth inhibitory effect of SPATA12 on UV-C irradiated cells

**Figure 8:**
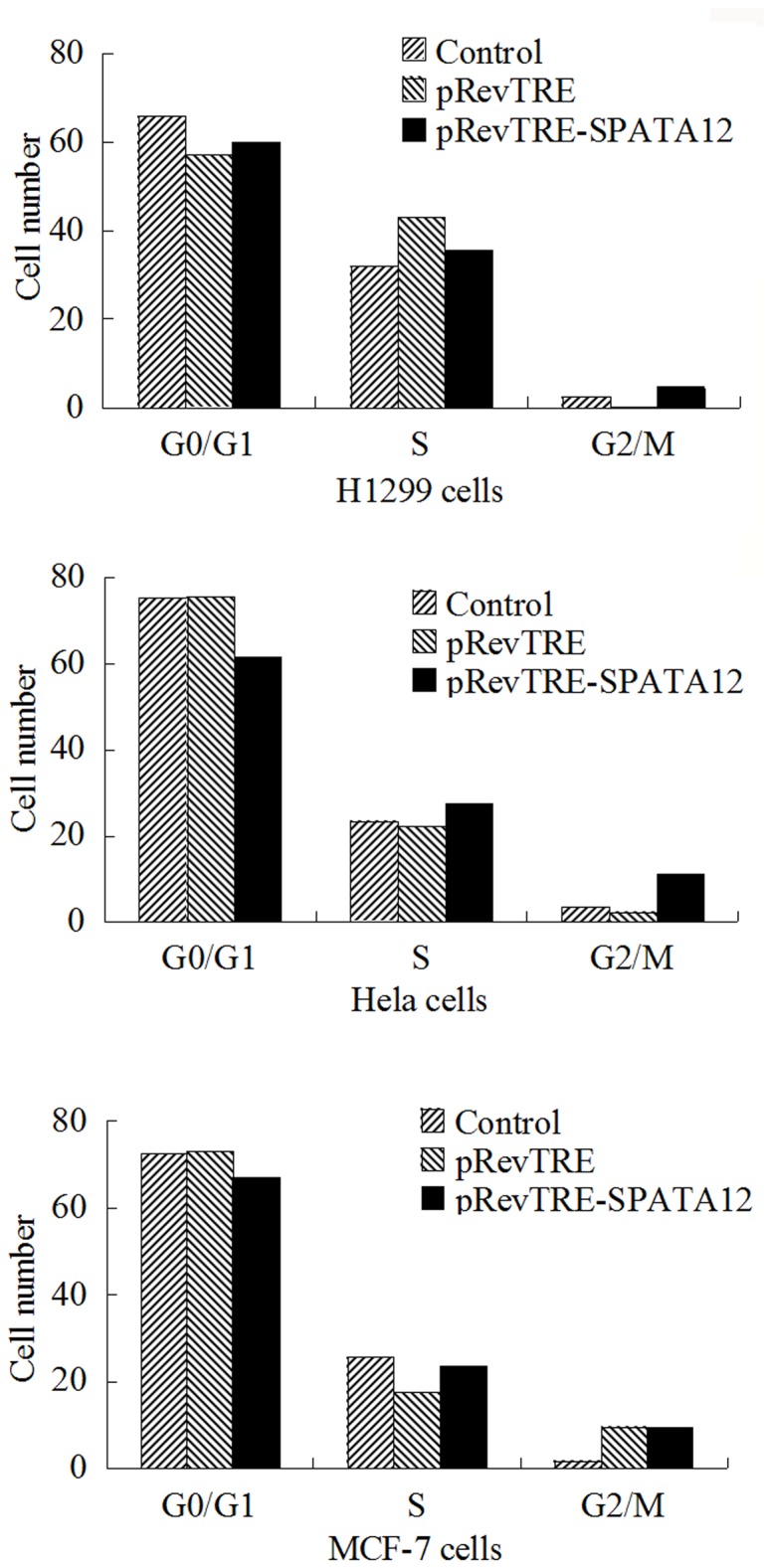
SPATA12 may affect cell cycle progression in a p53-dependent manner after UV-C irradiation. pRevTRE-SPATA12 and the empty vector pRevTRE were transfected into H1299 (p53-null), HeLa (p53 functional deletion) and MCF-7 (p53 wild type) cells. Cell lines were irradiated with 600 J/m^2^ UV-C and harvested for FCM analysis.

**Figure 9:**
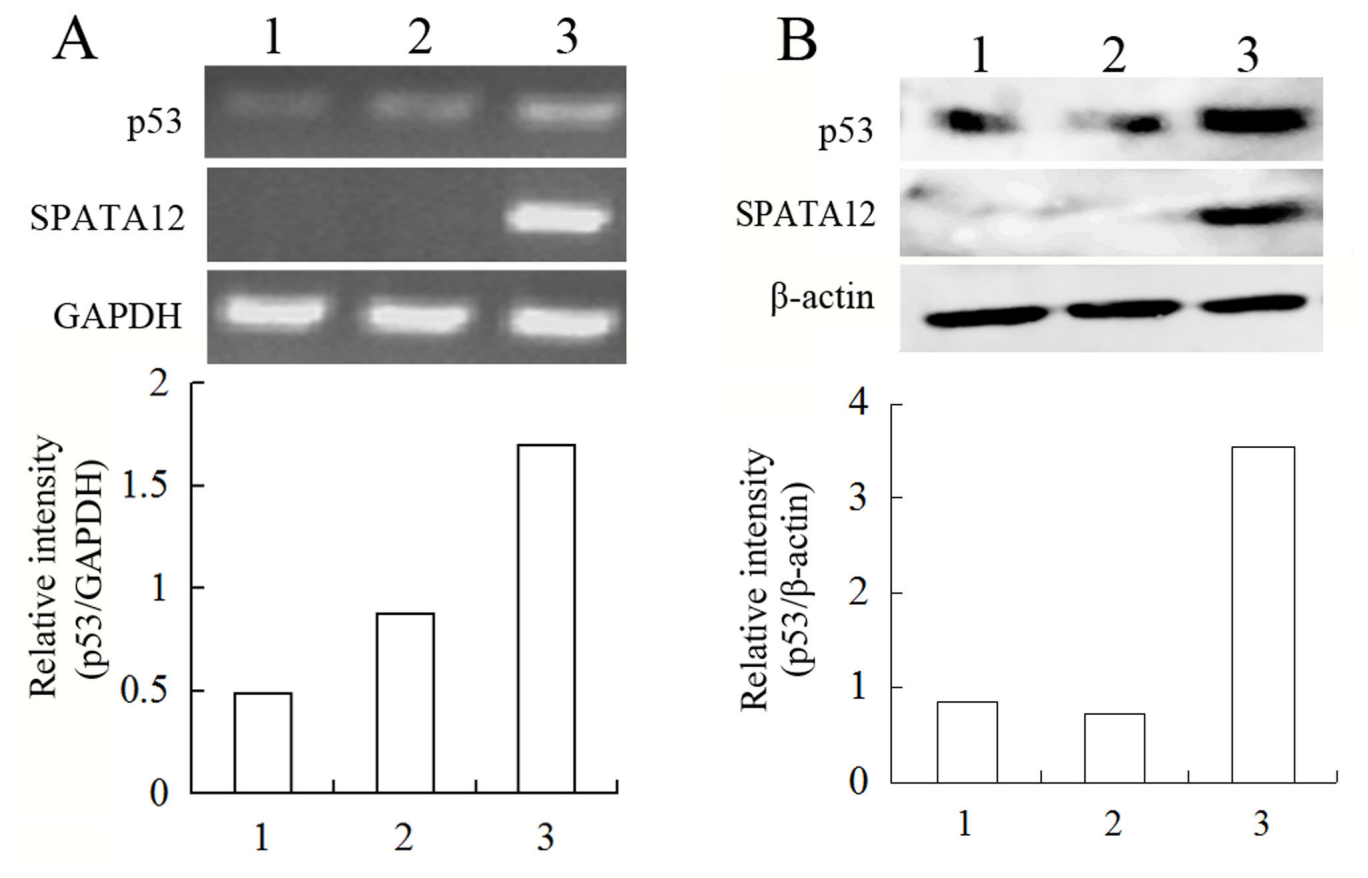
SPATA12 up-regulates the expression of p53 at both mRNA and protein level. A: The mRNA level of *p53* in MCF-7 cells was detected by RT-PCR. *GAPDH* was used for normalization control. B: The protein level of p53 in MCF-7 cells was detected by western blot. β-actin was used as normalization control. 1: Non-transfected group; 2: pRevTRE-transfected group; 3: pRevTRE-SPATA12-transfected group.

 Having demonstrated the involvement of SPATA12 in the DDR, we further sought to determine the mechanism. We focused on p53 signaling because it has been shown to have a direct and critical impact on DNA damage. SPATA12 was transfected into H1299 (p53 null), HeLa (p53 functional deletion) and MCF-7 cells (p53 wt) and cells were UV-C irradiated at 600 J/m^2^ to ascertain whether the growth inhibitory effect of SPATA12 on cells links to p53. Analysis of cell cycle distribution by FCM showed that there were no changes between the SPATA12 and empty vector control groups in H1299 cells. A delay at G1/S cell cycle transition was observed in the SPATA12 groups compared with empty vector control groups in both HeLa and MCF-7 cells (Figure 8), although the S phase arrest wasn’t significant statistically. Moreover, we found that SPATA12 could promote p53 expression at both mRNA (Figure 9A) and protein level (Figure 9B) in MCF-7 cells. These data suggested there are some biological association between SPATA12 and p53 and we speculated that p53 may involve in inhibition of SPATA12 on cell proliferation after DNA damage. 

## Discussion

SPATA12 is a newly identified human spermatogenesis-associated gene, which is specifically expressed in the germ cells of human adult testis and might play a critical role in spermatogenesis and tumorigenesis[12]. To further study the physiological function of SPATA12, we used a yeast two-hybrid system to search for proteins that interact with SPATA12 and identified CHD2. BiFC and subcellular localization assays further indicated a potential interaction of SPATA12 and CHD2 in nuclei, suggesting the probability that biologically, SPATA12 has a functional association with the CHD2 protein. 

The CHD family of proteins is characterized by the presence of chromo (chromatin organization modifier) domains and SNF2-related helicase/ATPase domains [15]. As a member of the CHD family, the CHD2 protein is known to directly function in the regulation of gene expression, chromatin remodeling and genomic stability via its chromatin specific interactions and activities[5-7]. Regarding the interaction between SPATA12 and CHD2, our hypothesis is that the testis-specific gene SPATA12 might be involved in the DNA damage response. Genetic evidence is now indicating that environmental agents such as UV radiation can interact with the male genome, causing specific types of genetic changes to sperm that affect fertility [16]. Some spermatogenesis-associated DNA damage repair genes have been identified, including *OGG1*, *XRCC1*, *ERCC2*, *ERCC1*, *BRCA1* and *BRCA2*, which are highly expressed in the testis, especially in meiotic spermatocytes[17]. In this study, SPATA12 was induced at both mRNA and protein levels in UV-C-irradiated cells, suggesting that SPATA12 might have a role in DNA damage. To further explore the effect of SPATA12 on cells undergoing DNA damage, colony formation and HCR assays was performed and demonstrated that SPATA12 may induce the inhibition of cell proliferation after UV-C radiation exposure, suggesting the involvement of SPATA12 in DDR and promoting us to ascertain how SPATA12 senses and/or responds to DNA damage. 

The cellular response to UV radiation involves multiple and specific signal transduction pathways and transcription factors [18-23]. It is known that the activation of intracellular signaling pathways in response to UV radiation induces various transcription factors, such as AP-1 and p53, and their subsequent modulation of gene expression [18]. Previous reports suggested that AP-1 is mainly composed of JUN, FOS and ATF dimers, which are thought to be involved in the regulation of DNA repair and implicated in maintaining genomic stability and cell survival by mediating gene expression in response to UV-C radiation [24,25]. The effects are generally mediated through the activation and binding of AP-1 dimers to target DNA consensus sequences. Interestingly, an AP-1 binding site was found in the *SPATA12* promoter region in our previous study, and we demonstrated that AP-1 as an activator plays a role in the regulation of *SPATA12* promoter activity [14]. Thus, we wondered whether the *SPATA12* activity induced by UV-C radiation might be through the AP-1 binding site. Using constructs containing the *SPATA12* promoter upstream of a luciferase reporter gene (AP-1 binding site included), we evidenced an association between the AP-1 binding site and transcriptional activity of the *SPATA12* promoter in response to UV-C radiation and verified that the AP-1 involves in the transcriptional regulation of *SPATA12* during UV-C induced DNA damage. 

In previous studies, we observed that the expression of SPATA12 results in a delay at the G1/S cell cycle transition [12,13]. It is known that a master regulator of passage from G1 to S is the p53-p21 pathway, and the p53 gene is a major target for UV-induced mutations [9]. In addition, CHD2, the interacting protein of SPATA12, was shown to affect the transcriptional activity of p53 in DNA damage signaling [5-7]. Thus, we hypothesized that the inhibition of cell cycle progression associated with the induction of SPATA12 might also be associated with p53 signaling in response to DNA damage stress. Three cell lines, including H1299 (p53 null), MCF-7 (p53 wt) and HeLa (p53 functional deletion), were used to verify this hypothesis. SPATA12-transfected H1299, MCF-7 and HeLa cells were UV-C irradiated, and FCM results suggested that there are some biological association between SPATA12 and p53. Importantly, we also found that SPATA12 could up-regulate p53 expression at both mRNA and protein levels, suggesting that p53 involve in growth inhibitory effects of SPATA12 on cells during DNA damage process.

In conclusion, we observed that SPATA12 may interact with CHD2 in the nucleus. During DNA damage process, AP-1 involves in the transcriptional up-regulation of *SPATA12* in response to UV-C radiation and p53 involves in growth inhibitory effects of SPATA12 on UV-C irradiated cells.

## Materials and Methods

### Yeast two-hybrid screening system

To generate a bait construct, the open reading frame (ORF) fragment of *SPATA12* was amplified by PCR from a human testis cDNA library (Clontech) using the following primers: 5' –CAG GAATTCATGTCCAGTTCTGCTCTGACT-3' (forward, *EcoR*I underscored) and 5' –CAGTTA GTCGACTTACAGATGTGTGTGTATGTG-3' (reverse, *Sal*I underscored) and inserted into the pGBKT7 vector (Clontech). The human testis cDNA library was inserted into the pGADT7-Rec vector (Clontech) as the hunter construct. The bait plasmid was transformed into the yeast strain AH109. The AH109 yeast containing pGBKT7/SPATA12 was then cultured on SD/-Trp and SD/-His/-Trp/-Ade to exclude autonomous transcriptional activity. AH109 yeast containing pGBKT7/53 was used as a positive control and AH109 yeast cells transformed with pGBKT7 were used as the negative control. The transformed AH109 yeast was mated with Y187 yeast (containing the human testis cDNA library). Secondary screening was based on further activation of the β-galactosidase reporter gene and was determined using blue/white colony screening. After being reintroduced into yeast AH109 cells and sequenced to verify expression of the ORF, plasmids were isolated from the positive yeast colonies and transformed into *E. coli* strain JM109 for further study.

### Cell Culture, Transient Transfections and UV-C Radiation

 The human tumor cell lines HeLa (ATCC CCL2), MCF-7 (ATCC HTB22) and NCI-H1299 (ATCC CRL-5803^TM^) were maintained in RPMI 1640 medium (Hyclone) supplemented with 10% fetal calf serum (Hyclone) and containing 100 units/ml of penicillin and 100 μg/ml streptomycin in a humidified atmosphere with 5% CO_2_ at 37 °C. 

For transient transfections, cells were seeded in a modular incubator chamber 24 h before transfection. All transfections were performed with TurboFect^TM^ *in vitro* Transfection Reagent (Fermentas), according to the manufacturer’s instructions.

For UV-C irradiation, cells were directly exposed to a 30 cm ultraviolet lamp (UV-C, 254 nm) at does of 600 J/m^2^ (equivalent to rate of 0.1667 J/m^2^ per second) as measured by a quantum photometer.

### Bimolecular fluorescence complementation (BiFC) assay

 To construct recombinant BiFC plasmids, the ORF fragments of *CHD2* and *SPATA12* were respectively amplified by PCR using the following primers: 5'-CGCGGATCCgccaccATGATGA GAAATAAGGACAAAAGC-3' (forward, *Bam*HI included) and 5'-CGGGGTACCCTTGCA CCAGGAATGAGCTAG-3' (reverse, *Kpn*I included) for *CHD2*; and 5'-CGCGGATCCgccaccATGTCCAGTTCTGCTCTGACT-3' (forward, *Bam*HI included) and 5'-CGGGGTACCCAGATGTGTGTGTATGTGTGTGTT-3' (reverse, *Kpn*I included) for *SPATA12*. Fragments of YFP from the PA7-YFP vector were truncated at residue 155 (designated YN and YC) as previously described [26], using the following primers: 5-'CGGGGTACCCGCTCCATCGCCACGATGGTGAGCAAGGGCGAG-3' (forward, *Kpn*I included) and 5'-CCGGAATTCTTAGGCCATGATATAGACGTTGT-3' (reverse, *EcoR*I included) for *YN*; and 5'-CGGGGTACCCGCCCGGCCTGCAAGATCCCGAACG ACCTGAAACAGAAGGTCATGAACCACGACAAGCAGAAGAACGGCAT-3' (forward, *Kpn*I included) and 5'-CCGGAATTCTTACTTGTACAGCTCGTCCATGC-3' (reverse, *EcoR*I included) for *YC*. In each of the above primer sequences, underlined letters represent nucleotides inserted to introduce restriction sites and italicized letters represent the RSIAT and RPACKIPNDLKQKVMNH linker sequences encoding the GGGS linker peptide that facilitates the association between the target proteins and YN/YC, as previously described [26]. Lower-case letters represent Kozak translation initiation sequences and an ATG start codon for proper initiation of translation [27]. The YN fragment was connected to the C-terminus of CHD2, and the YC fragment was connected to the C-terminus of SPATA12. The connected fragments described above were inserted into the pcDNA3.1(+) vector to generate pcDNA3.1(+)-CHD2-YN and pcDNA3.1(+)-SPATA12-YC. All constructs were verified by sequencing (Sanggon Biotech Co., Ltd). 

For the BiFC assay, cells at 80% confluence were co-transfected with a mixture of 2 μg pcDNA3.1(+)-CHD2-YN and 2 μg pcDNA3.1(+)-SPATA12-YC using TurboFect^TM^ *in vitro* Transfection Reagent. Cells were maintained in 5% CO_2_ and 95% humidified air atmosphere at 37 °C for 24h, and then cells were cultured at 30 °C for 24h. Finally, cells were analyzed using a laser scanning confocal microscope (Leica SP 2 series).

### Subcellular localization

For the pEGFP-SPATA12 construct, the ORF fragment of *SPATA12* was amplified by PCR and subcloned into the pEGFP-C2 vector (Clontech) using the following primers: 5'-CCCAAGCTTCATGTCCAGTTCTGCTCTGACT-3' (forward, *Hind*III included) and 5'-CGCGGATCCTTACAGATGTGTGTGTATGTG-3' (reverse, *BamH*I included). For the pDsRed-CHD2 construct, the ORF fragment of *CHD2* was amplified by PCR and subcloned into pDsRed-C1 vector (Clontech) using the following primers: 5′-CGGGGTACCA TGATGAGAAATAAGGACAAAAGC-3′ (forward, *Kpn*I included) and 5′-CGCGGATCCCTAC TTGCACCAGGAATGAGCTA G-3′ (reverse, *BamH*I included). All constructs were verified by sequencing (Sanggon Biotech Co., Ltd).

Cells at 75% confluence were co-transfected with a mixture of 2 μg pEGFP-SPATA12 and 2 μg pDsRed-CHD2 using TurboFect^TM^ *in vitro* Transfection Reagent. Cells were then analyzed using a laser scanning confocal microscope (Leica SP 2 series). equipped with a cooled CCD camera with a 488-nm and a 563-nm excitation beam. 

### Cell survival assay

 Cells were plated on 96-well plates (5,000 cells /well) the day before UV-C irradiation. The cells were left untreated or were treated with the indicated doses of UV-C radiation. Cells were treated with MTT (20 μl of 5 mg/ml) for 4 h at 37 °C for the formation of purple formazan crystals. Crystals were solubilized in dimethyl sulfoxide (DMSO) for 10 min, and the absorbance was measured in a 96-well platereader (Bio-Rad) at 570 nm. Experiments were performed in quadruplicate, and results were normalized compared to control levels.

### Hochest-PI double staining

 After UV-C irradiation, cells were collected and fluorescence intensity was investigated by Hochest-PI staining using an inverted fluorescence microscope (Nikon). Non-irradiated cells were used as the control group. 

### Flow cytometry analysis

 UV-C-irradiated cells were collected by trypsinization and pelleted by centrifugation at 800 × *g* for 5 min. Cell pellets were resuspended and fixed in 70% cold ethanol overnight. After being washed with PBS, cells were mixed with PI staining solution (final concentration of 50 μg/mL) for 30 min at room temperature in the dark. The apoptotic peak was investigated and apoptotic rate was calculated by flow cytometry. Non-irradiated cells were used as the control group. 

### Neutral comet assay (single cell gel electrophoresis assay)

 The neutral comet assay was performed as previously described to detect DNA damage in UV-C-irradiated cells [5,6]. Images were obtained and tail moments were determined using CASP software. The tail moment is considered the most sensitive parameter of the comet assay; it represents the integrated value of the fluorescence intensity multiplied by migration distance and is proportional to the level of damaged DNA. For each experiment, 50 cells were scored from replicate slides (100 cells total) and subsequently pooled.

### Colony formation assay

 Cells were transfected with pRevTRE-SPATA12, UV-C-irradiated, collected by trypsinization and seeded in 6-well plates at 50 cells per plate. The plates were incubated for 14 days in a humidified atmosphere with 5% CO_2_ at 37 °C. The plates were fixed with carbinol, stained with Giemsa solution and colonies were counted. Non-irradiated cells were used as the control group.

### Preparation of total RNA, RT-PCR and SYBR-green real-time RT-PCR

 Total RNA was isolated by TRIzol reagent (Invitrogen) and 3μg of total RNA were reverse transcribed using M-MLV Reverse Transcriptase (Promega). The following sense (S) and antisense (AS) primer sequences were used: *SPATA12*-S, 5'-TCACCTTCCCCTCATCTCCC-3'; *SPATA12*-AS, 5'-TTTCACGCTTGTCCACTTTCAC-3'; *CHD2*-S, 5'-GAAGCTTCGGGTTCAG ACTCA-3'; *CHD2*-AS, 5'-TTCGACTCGCTGCCATGTC-3'; *p53*-S, GCTTTGAGGTGCGTGTT TGT-3'; *p53*-AS, 5'-TTGGGCAGTGCTCGCTTA-3'. Human *β-actin* or *GAPDH* mRNA levels were used for normalization of SYBR-green real-time RT-PCR results, and the primers are as follows: *β-actin*-S, 5'-AGATCATGTTTGAGACCTTCAACAC-3'; *β-actin*-AS, 5'-GGAGCAATGATCTTGATCTTCATTG-3'; *GAPDH*-S, 5'-GACCCCTTCATTGACCTCAA-3'; *GAPDH*-AS, 5'-GCATGGACTGTGGTCATGAGT-3'. The mRNA levels of *SPATA12*, *CHD2* and *p53* were monitored by real-time PCR as described previously [27].

### Western blot

 Cell pellets were lysed and extracted in 500 μl of RIPA Lysis Buffer (50 mM Tris (pH 7.4), 150 mM NaCl, 0.1% SDS, 1% Nonidet P-40, 0.5% sodium deoxycholate) containing freshly added 1 mM Phenylmethanesulfonyl Fluoride (PMSF) and 2.5 μl of Protease Inhibitor Cocktail Set iii (Calbiochem). The protein concentration was determined using a BCA-protein quantification assay, and western blot was performed as previously described [28]. The primary antisera included antibodies specific for p53 (Santa Cruz), p-p53(Ser15) (Santa Cruz), p-p53(Ser33) (Cell Signaling), β-actin (BOSTER), CHD2 (Cell Signaling) and SPATA12 (Beijing Biosynthesis Biotech Co.,LTD). The protein expression levels were quantitated using Quantity One software (Bio-Rad).

### Construction and transfection of AP-1 decoy oligodeoxynucleotides

 Transfection of cells with short double-stranded synthetic DNA molecules that contain a transcription factor binding site, known as decoy oligodeoxynucleotides(ODNs), has been proposed as a novel approach in vitro and in vivo for the study of gene regulation [29,30]. Synthetic, double-stranded, HPLC purified, phosphorothioate ODN specific for AP-1, together with matching mutated ODN as a control, were purchased from Takara Biotechnology Co., Ltd. as follows: AP-1 decoy ODNs, 5'-AG*CTTGTGAGTCAGAAG*C*T-3' and 3'-T*C*GAACACTCAGTCTTC*GA-5'; mismatched decoy ODNs, 5'-AG*CTTGAATCTCAGAAG*C*T-3' and 3'-T*C*GAACTTAGAGTCTTC*GA-5' (the consensus and the corresponding mutated sequences are underlined, and * denotes a phosphorothioate modification) [31,32]. Double-stranded 19-mer ODNs (5 μM) were annealed by Takara Biotechnology Co., Ltd.

 Approximately 10^5^ cells/well were plated in 24-well plates one day prior to transfection. When 60-70% confluent, 100 nM AP-1 ODNs were transfected using TurboFect^TM^ *in vitro* Transfection Reagent (Fermentas). After 4 h of incubation at 37 °C in a 5% CO_2_ incubator, 500 ml normal growth media was added to each well. Cellular extracts were prepared 24 h post-transfection, as described below. Each experiment included transfection with both wild-type and mutant AP-1 decoy ODNs, with untransfected cells as an additional control. The same protocol was used for all cell lines. 

### Dual luciferase reporter gene assay and host cell reactivation assay

The recombinant *SPATA12* promoter reporter plasmid pGL3 -136/302 (wild-type AP-1 binding site), deletion construct pGL3 -136/302 (132-134 of the AP-1 binding site deleted) and point mutation construct pGL3 -136/302 (132-134 of the AP-1 binding site mutated) were constructed by our group in a previous study [14]. Transfections were performed using TurboFect^TM^ *in vitro* Transfection Reagent (Fermentas), and a plasmid encoding renilla luciferase (Promega) was used as a control for transfection efficiency. Forty hours after transfection, cells were irradiated and harvested, and both firefly and renilla luciferase activities were determined using a Dual-Luciferase Reporter Assay System (Promega) on a Modulus^TM^ luminometer (Turner biosystems). All reporter assays were performed in triplicate. Non-irradiated cells were used as the control group.

 For the host cell reactivation (HCR) assay, the firefly luciferase assay was carried out as previously described [33]. Briefly, the pGL3-control plasmid (50 mg/mL) was UV-C irradiated at 30 J/m^2^ in a 24-well plate on ice. UV-C-induced damage was verified by PCR with a forward primer of 5'-CTGAAGTCCGCCAGTTGCT-3' and a reverse primer of 5'-TTGCCTAATGGTCCCTAAAGTC-3'. The irradiated or non-irradiated control plasmids (2 μg) were then transfected into HeLa cells using Lipofectamine (Invitrogen). The plasmid encoding renilla luciferase was used as a control for transfection efficiency. Forty hours after transfection, cells were harvested and both firefly and renilla luciferase activities were determined using the Dual-Luciferase Reporter Assay System (Promega) on a Modulus^TM^ (Turner biosystems).

### Statistical analysis

Statistical analysis was conducted using Student’s *t*-test. The *p*-values ≤0.05 were considered significant.

## Supporting Information

Figure S1 **Establishment of a cellular DNA damage model induced by UV-C radiation.** A: The effect of UV-C radiation on HeLa cell viability was detected by MTT assay. The representative experiment shows the mean ± standard error with the significant difference between each dose of UV-C and the control evaluated using Student’s *t*-test, **p< 0.01. B and C: Analysis of the effects of UV-C radiation on cell cycle distribution and apoptosis were detected by FCM assay. D: The morphological changes of the apoptotic cell were observed by Hoechst-PI double staining (×400).
(TIF)

Figure S2 **The interaction of SPATA12 and CHD2 in UV-C induced DNA damage condition by BiFC assay.** Cells were co-transfected with pcDNA3.1(+)-CHD2-YN and pcDNA3.1(+)-SPATA12-YC plasmids and then irradiated with 600 J/m^2^ UV-C. And yellow signal represents interaction of SPATA12 and CHD2 in nuclei in DNA damage condition (×400).
(TIF)

Figure S3 **The expression of *SPATA12* were induced by UV-C radiation at mRNA level.** The mRNA levels of *SPATA12*, *CHD2* and p53 in UV-C radiated cells were detected by real time RT-PCR, respectively. *GAPDH* was used as normalization control.
(TIF)

## References

[1] Li H, Mitchell JR, Hasty P (2008) DNA double-strand breaks: a potential causative factor for mammalian aging? Mech Ageing Dev 129(7-8): 416-424. doi:10.1016/j.mad.2008.02.002. PubMed: 18346777. 18346777 PMC2517577

[2] Pandita TK, Richardson C (2009) Chromatin remodeling finds its place in the DNA double-strand break response. Nucleic Acids Res 37(5): 1363-1377. doi:10.1093/nar/gkn1071. PubMed: 19139074. 19139074 PMC2655678

[3] Costelloe Thomas, FitzGerald Jennifer, Murphy Niall J, Flaus Andrew, Lowndes Noel F (2006) Chromatin modulation and the DNA damage response. Exp Cell Res. 312: 2677-2686. doi:10.1016/j.yexcr.2006.06.031. PubMed: 16893724. 16893724

[4] van Attikum H, Gasser SM (2009) Crosstalk between histone modifications during the DNA damage response .Trends Cell Biol 19(5): 207-217. doi:10.1016/j.tcb.2009.03.001. PubMed: 19342239. 19342239

[5] Rajagopalan S, Nepa J, Venkatachalam S (2012) Chromodomain helicase DNA-binding protein 2 affects the repair of X-ray and UV-induced DNA damage. Environ Mol Mutagen 53(1): 44-50. doi:10.1002/em.20674. PubMed: 22223433. 22223433

[6] Nagarajan P, Onami TM, Rajagopalan S, Kania S, Donnell R et al. (2009) Role of chromodomain helicase DNA-binding protein 2 in DNA damage response signaling and tumorigenesis. Oncogene. 28(8): 1053-1062. doi:10.1038/onc.2008.440. PubMed: 19137022. 19137022 PMC2648865

[7] Marfella CG, Ohkawa Y, Coles AH, Garlick DS, Jones SN et al. (2006) Mutation of the SNF2 family member Chd2 affects mouse development and survival. J Cell Physiol 209(1): 162-171. doi:10.1002/jcp.20718. PubMed: 16810678. 16810678

[8] Lavin MF, Gueven N (2006) The complexity of p53 stabilization and activation. Cell Death Differ 13(6): 941-950. doi:10.1038/sj.cdd.4401925. PubMed: 16601750. 16601750

[9] Liu Y, Kulesz-Martin M (2001) p53 protein at the hub of cellular DNA damage response pathways through sequence-specific and non-sequence-specific DNA binding . Carcinogenesis. Volumes 6: 851-860. 10.1093/carcin/22.6.851 11375889

[10] Sakaguchi K, Herrera JE, Saito S, Miki T, Bustin M et al. (1998) DNA damage activates p53 through a phosphorylationacetylation cascade. Genes Dev 12(18): 2831-2841. doi:10.1101/gad.12.18.2831. PubMed: 9744860. 9744860 PMC317174

[11] Farnebo M, Bykov VJ, Wiman KG (2010) The p53 tumor suppressor: a master regulator of diverse cellular processes and therapeutic target in cancer. Biochem Biophys Res Commun 396(1): 85-89. doi:10.1016/j.bbrc.2010.02.152. PubMed: 20494116. 20494116

[12] Dan L, Lifang Y, Guangxiu L (2007) Expression and possible functions of a novel gene SPATA12 in human testis. J Androl 28(4): 502-512. doi:10.2164/jandrol.106.001560. PubMed: 17251597. 17251597

[13] Liu Z, Lin Y, Liu X, Yu W, Zhang Y et al. (2012) Experimental study of inhibition of tumor cell proliferation by a novel gene SPATA12. Zhong NAN Xue Xue Bao Yi Xue Ban. 37(3): 222-227. 10.3969/j.issn.1672-7347.2012.03.002 22561491

[14] Li D, Lin Y, Liu Z, Zhang Y, Rong Z et al. (2012) Transcriptional regulation of human novel gene SPATA12 promoter by AP-1 and HSF. Gene. 511(1): 18-25. doi:10.1016/j.gene.2012.08.047. PubMed: 22981541. 22981541

[15] Marfella Concetta GA, Imbalzano Anthony N (2007) The Chd Family of Chromatin Remodelers. Mutat Res 618(1-2): 30-40. doi:10.1016/j.mrfmmm.2006.07.012. PubMed: 17350655. 17350655 PMC1899158

[16] Olsen AK, Lindeman B, Wiger R, Duale N, Brunborg G (2005) How do male germ cells handle DNA damage? Toxicol Appl Pharmacol 207(2 Suppl): 521-531. doi:10.1016/j.taap.2005.01.060. PubMed: 16051290. 16051290

[17] Aitken RJ, Baker MA (2006) Oxidative stress,sperm survival and fertility control.Mol Cell Endocrinol 250: 66-69. doi:10.1016/j.mce.2005.12.026. PubMed: 16412557. 16412557

[18] López-Camarillo C, Ocampo EA, Casamichana ML, Pérez-Plasencia C, Alvarez-Sánchez E et al. (2012) Protein Kinases and Transcription Factors Activation in Response to UV-Radiation of Skin: Implications for Carcinogenesis. Int J Mol Sci 13(1): 142-172. PubMed: 22312244. 22312244 10.3390/ijms13010142 PMC3269678

[19] Sachsenmaier C, Radler-Pohl A, Zinck R, Nordheim A, Herrlich P et al. (1994) Involvement of growth factor receptors in the mammalian UVC response. Cell. 78: 963-972. doi:10.1016/0092-8674(94)90272-0. PubMed: 7923365. 7923365

[20] Zhang Y, Dong Z, Bode AM, Ma WY, Chen N et al. (2001) Induction of EGFR-dependent and EGFR-independent signaling pathways by ultraviolet A irradiation. DNA Cell Biol 20: 769-779. doi:10.1089/104454901753438589. PubMed: 11879570. 11879570

[21] Bode AM, Dong Z (2003) Mitogen-activated protein kinase activation in UV induced signal transduction. Sci STKE. 167:RE2 10.1126/stke.2003.167.re2 12554854

[22] Zhong JL, Yang L, Lü F, Xiao H, Xu R et al. (2011) UVA, UVB and UVC Induce Differential Response Signaling Pathways Converged on the eIF2α phosphorylation. Photochem Photobiol 87: 1092-1104. doi:10.1111/j.1751-1097.2011.00963.x. PubMed: 21707633. 21707633

[23] Rastogi RP, Richa, Kumar A, Tyagi MB, Sinha RP (2010) Molecular Mechanisms of Ultraviolet Radiation-Induced DNA Damage and Repair. J Nucleic Acids, 2010: 592980 PubMed: 21209706 10.4061/2010/592980 PMC3010660 21209706

[24] Huang C, Li J, Chen N, Ma W, Bowden GT et al. (2000) Inhibition of atypical PKC blocks ultraviolet-induced AP-1 activation by specifically inhibiting ERKs activation. Mol Carcinog 27: 65-75. doi:10.1002/(SICI)1098-2744(200002)27:2. PubMed: 10657899. 10657899

[25] Zhou H, Gao J, Lu ZY, Lu L, Dai W et al. (2007) Role of c-Fos/JunD in protecting stress-induced cell death. Cell Prolif 40(3): 431-444. doi:10.1111/j.1365-2184.2007.00444.x. PubMed: 17531086. 17531086 PMC6496388

[26] Kerppola TK (2006) Design and implementation of bimolecular fluorescence complementation (BiFC) assays for the visualization of protein interactions in living cells. Nat Protoc 1: 1278–1286. doi:10.1038/nprot.2006.201. PubMed: 17406412. 17406412 PMC2518326

[27] Kozak M (1991) An Analysis of Vertebrate mRNA Sequences: Intimations of Translational Control. J Cell Biol 115: 887-903. doi:10.1083/jcb.115.4.887. PubMed: 1955461. 1955461 PMC2289952

[28] Li ZX, Wang TT, Wu YT, Xu CM, Dong MY et al. (2008) Adriamycin induces H2AX phosphorylation in human spermatozoa. Asian J Androl 10(5): 749-757. doi:10.1111/j.1745-7262.2008.00400.x. PubMed: 18645678. 18645678

[29] Dzau VJ (2002) Transcription factor decoy. Circ Res, 90: 1234–1236. doi:10.1161/01.RES.0000025209.24283.73. PubMed: 12089058. 12089058

[30] Morishita R, Higaki J, Tomita N, Ogihara T (1998) Application of transcription factor ‘decoy’ strategy as means of gene therapy and study of gene expression in cardiovascular disease. Circ Res, 82: 1023–1028. doi:10.1161/01.RES.82.10.1023. PubMed: 9622154. 9622154

[31] Ahn JD, Morishita R, Kaneda Y, Lee KU, Park JY et al. (2001) Transcription factor decoy for activator protein-1 (AP-1) inhibits high glucose- and angiotensin II-induced type 1 plasminogen activator inhibitor (PAI-1) gene expression in cultured human vascular smooth muscle cells. Diabetologia. 44(6): 713-720. doi:10.1007/s001250051680. PubMed: 11440364. 11440364

[32] Ahn JD, Morishita R, Kaneda Y, Lee SJ, Kwon KY et al. (2002) Inhibitory effects of novel AP-1 decoy oligodeoxynucleotides on vascular smooth muscle cell proliferation in vitro and neointimal formation in vivo. Circ Res 90(12): 1325-1332. doi:10.1161/01.RES.0000023200.19316.D5. PubMed: 12089071. 12089071

[33] Qiao Y, Spitz MR, Guo Z, Hadeyati M, Grossman L et al. (2002) Rapid assessment of repair of ultraviolet DNA damage with a modified host-cell reactivation assay using a luciferase reporter gene and correlation with polymorphisms of DNA repair genes in normal human lymphocytes. Mutat Res 509: 165-174. doi:10.1016/S0027-5107(02)00219-1. PubMed: 12427537. 12427537

