# Supplementary figures and images for: SPATA12 and Its Possible Role in DNA Damage Induced by Ultraviolet-C
Source: PLoS One. 2013 Oct 18;8(10):e78201. doi: 10.1371/journal.pone.0078201 (PMC3799742; doi:10.1371/journal.pone.0078201)

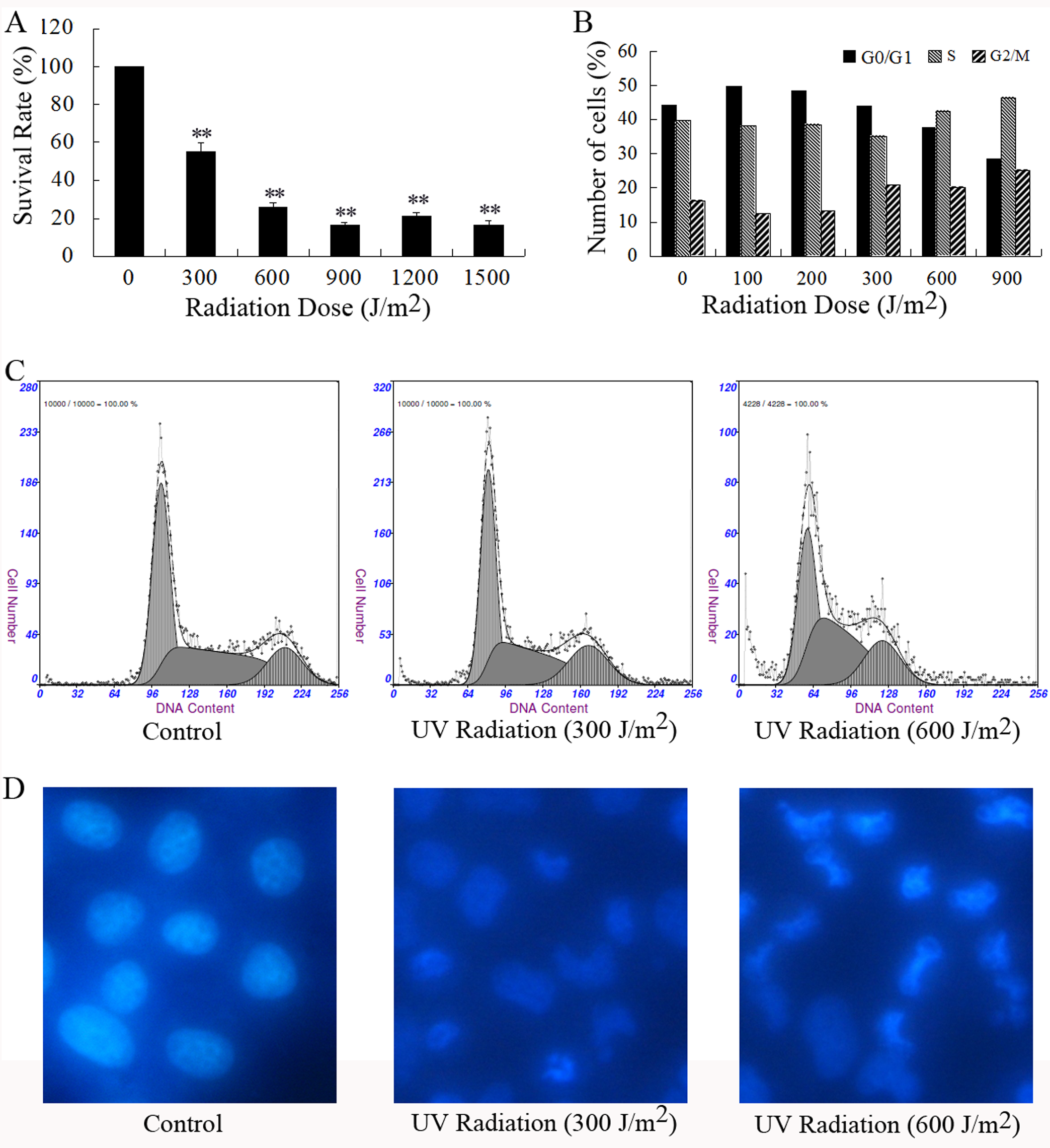

Supplement: Figure S1 — Establishment of a cellular DNA damage model induced by UV-C radiation. A: The effect of UV-C radiation on HeLa cell viability was detected by MTT assay. The representative experiment shows the mean ± standard error with the significant difference between each dose of UV-C and the control evaluated using Student’s t-test, **p< 0.01. B and C: Analysis of the effects of UV-C radiation on cell cycle distribution and apoptosis were detected by FCM assay. D: The morphological changes of the apoptotic cell were observed by Hoechst-PI double staining (×400). (TIF) [file pone.0078201.s001.tif]

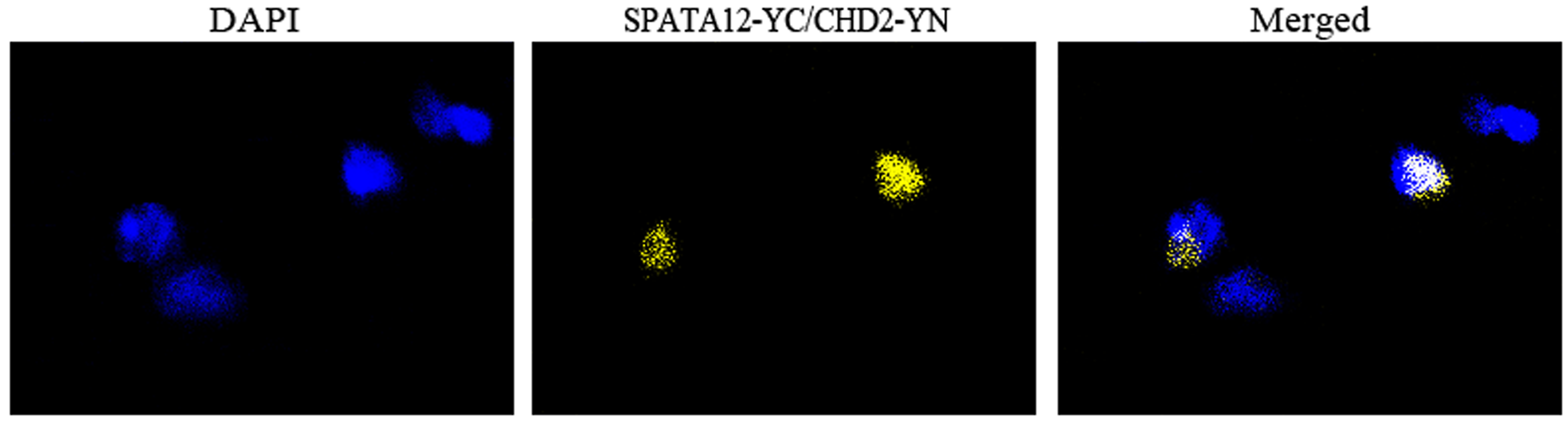

Supplement: Figure S2 — The interaction of SPATA12 and CHD2 in UV-C induced DNA damage condition by BiFC assay. Cells were co-transfected with pcDNA3.1(+)-CHD2-YN and pcDNA3.1(+)-SPATA12-YC plasmids and then irradiated with 600 J/m2 UV-C. And yellow signal represents interaction of SPATA12 and CHD2 in nuclei in DNA damage condition (×400). (TIF) [file pone.0078201.s002.tif]

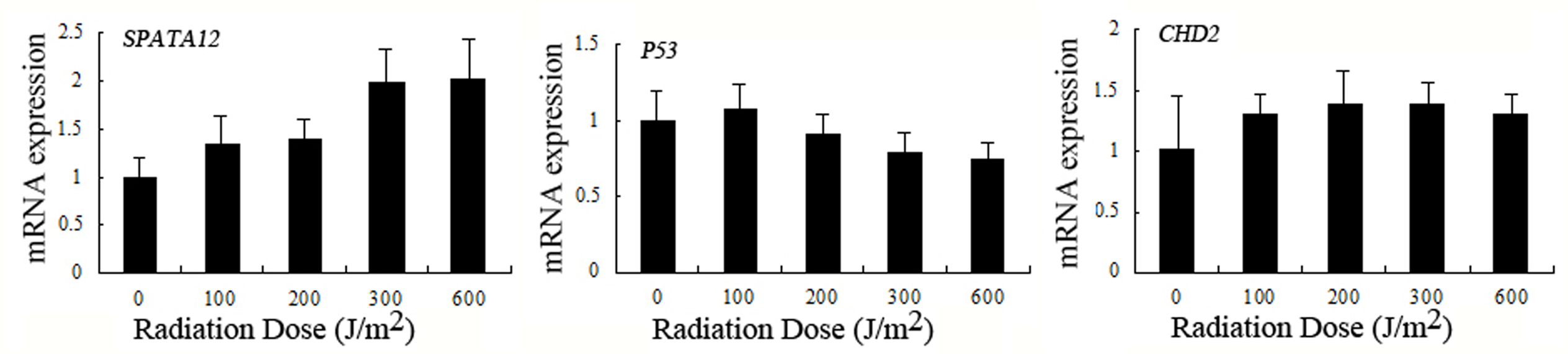

Supplement: Figure S3 — The expression of SPATA12 were induced by UV-C radiation at mRNA level. The mRNA levels of SPATA12, CHD2 and p53 in UV-C radiated cells were detected by real time RT-PCR, respectively. GAPDH was used as normalization control. (TIF) [file pone.0078201.s003.tif]
